# Bone Marrow Examination in the Diagnosis of Infectious Pathology: An Often Underutilized Diagnostic Tool

**DOI:** 10.7759/cureus.108330

**Published:** 2026-05-05

**Authors:** Monika Maharjan, Priyavadhana Balasubramanian, Arvind K Gupta, Neha Singh, Harish Chandra, Shalinee Rao

**Affiliations:** 1 Department of Pathology and Laboratory Medicine, All India Institute of Medical Sciences, Rishikesh, IND

**Keywords:** bone marrow hemophagocytosis, bone marrow morphology, diagnostic, infectious and parasitic diseases, laboratory hematology

## Abstract

Background: Bone marrow examination (BME) is one of the most important investigations in patients presenting with pyrexia of unknown origin (PUO) and cytopenias.

Purpose: This study aimed to assess the diagnostic yield of BME in infectious etiologies, determine their spectrum, and characterize the associated hematological and reactive marrow morphological changes.

Methods: It is a retrospective study, done on available data of all the infections diagnosed on the BME of six years, from January 2018 to December 2023. The clinical features and hematological findings were recorded. Cellularity of marrow, morphology of trilineage hematopoiesis, cell count, hemophagocytosis, iron stores, presence of organisms, and granulomas were evaluated.

Results: A total of 29 cases of infections were diagnosed, and the most common infection diagnosed was leishmaniasis in 19 (65.5%) cases, followed by tuberculosis and histoplasmosis seen in three (10.3%) cases each. Two (6.8%) cases showed infiltration of bone marrow by Hansen's disease, and one case each of cryptococcosis and disseminated *Mycobacterium avium *intracellulare infection. Anemia was the most common hematological finding, followed by thrombocytopenia, pancytopenia, and leukopenia. On BME, the prominence of histiocytes followed by erythroid hyperplasia and plasmacytosis was the most common reactive change.

Conclusion: BME is a simple, rapid, and economical diagnostic modality to diagnose infections. The presence of an organism has to be ruled out with bone marrow aspiration (BMA)/biopsy with anemia and plasmacytosis for PUO. Careful scrutiny of BMA slides is essential to identify soft clues that indicate an underlying infection. It plays a pivotal role in cases of PUO, particularly when other diagnostic modalities are inconclusive or when a rapid diagnosis is essential to enable timely management.

## Introduction

Bone marrow aspiration (BMA) and biopsy are simple, safe, and widely performed procedures for the diagnosis of hematological and nonhematological disorders. Bone marrow examination (BME) is one of the most important investigations in patients presenting with pyrexia of unknown origin (PUO) and cytopenias [1]. It provides relatively rapid information about the presence of an infectious etiology, along with information about associated hematologic abnormalities [2]. In cases of infections, BME may either show the specific etiological organisms or may reveal some nonspecific reactive changes which may suggest a high probability of a particular infection [3].

Routine Giemsa-stained BMA can reveal parasites like *Leishmania*, *Microfilaria*, and *Plasmodium*; fungi like *Histoplasma*, *Cryptococcus*, and certain viral inclusions. Addition of special stains like Ziehl-Neelsen (ZN), periodic acid-Schiff (PAS), Gomori's methenamine silver (GMS), and mucicarmine can further improve the sensitivity of BME. A bone marrow biopsy (BMB) can identify the presence of granulomas, aiding in the diagnosis of specific infectious diseases such as tuberculosis [1]. This study aimed to assess the diagnostic yield of BME in infectious etiologies, determine their spectrum, and characterize the associated hematological and reactive marrow morphological changes.

This article was previously presented as an E-Poster at the European Hematology Association Hybrid Congress 2024, held from June 13 to June 16 in Madrid, Spain, and as a poster in the 64th Annual Conference of the Indian Society of Haematology and Blood Transfusion, Hematocon, held from November 2 to November 5, 2023, in Bhubaneswar, Odisha.

## Materials and methods

Study design and setting

This was a retrospective descriptive study conducted in the Department of Pathology at a tertiary care center in North India over a period of six years, from January 2018 to December 2023.

Study population

All cases diagnosed with a definite infectious etiology on bone marrow aspirate and/or BMB and/or confirmed with special stains during the study period were included. Cases of noninfectious etiology were excluded from the study. Relevant clinical details, like age, gender, and presenting complaints, were retrieved from hospital medical records.

Morphological examination

BMA slides stained with Giemsa stain and BMB slides stained with hematoxylin and eosin were reviewed by two experienced pathologists independently. The following parameters were assessed: (a) overall cellularity of marrow and myeloid to erythroid ratio; (b) morphology and maturation of trilineage hematopoiesis; (c) presence and proportion of lymphocytes, plasma cells, eosinophils, and macrophages; (d) evidence of hemophagocytosis; (e) iron stores assessed on Perls' Prussian blue stain; and (f) presence of granulomas, whether necrotizing or nonnecrotizing, e.g., identification of intracellular organisms, whether seen intracellularly or extracellularly.

Special Stains

Special stains were also reviewed wherever performed on BMA smears and biopsy sections to confirm the infectious etiology. These stains included Ziehl-Neelsen stain (ZN) for acid-fast bacilli, *Mycobacterium tuberculosis*, Fite-Faraco stain for *Mycobacterium leprae, *and PAS and GMS stains for fungal organisms.

Data collection and statistical analysis

Data were entered into MS Excel (Microsoft Corporation, Redmond, Washington, United States) and analyzed using descriptive statistics. Continuous variables were summarized as median and range, calculated using built-in Excel functions. Categorical variables were expressed as frequencies and percentages.

Ethical considerations

The study was conducted following approval from the Institutional Ethics Committee, AIIMS/IEC/24/249. Patient data were anonymized and de-identified before analysis, and no identifying information was disclosed.

## Results

A total of 3,209 BMAs were examined during the study period, of which 29 cases (0.9%) were diagnosed with an infectious etiology on BME. The most common infection diagnosed was leishmaniasis in 19 (65.5%) cases, followed by tuberculosis and histoplasmosis in three (10.3%) cases each. Two (6.8%) cases showed Hansen's disease, and one case each had cryptococcal and *Mycobacterium avium* intracellulare complex (MAC) infection.

Clinical profile

The age of patients ranged from two to 68 years. The mean age was 30.9 + 17. There were 24 males (82.8%) and five females (17.2%). Fever was the most common presenting symptom observed in 28 cases (96.5%), followed by anorexia and weight loss. On clinical examination, splenomegaly was noted in 18 cases (62%), while hepatomegaly was present in 13 cases (44.8%).

Hematological findings

On complete blood count analysis, anemia was seen in all the cases, followed by thrombocytopenia in 27 (93.1 %), pancytopenia in 24 (82.8%), and neutropenia in 24 (82.8%) patients (Table 1). These findings indicate that cytopenias, particularly pancytopenia, are a prominent feature in patients with bone marrow infections. The median hemoglobin concentration was 6.8 gm/dl. The median total leukocyte count was 2080/cu.mm, whereas the median absolute neutrophil count was 780/cu.mm; the median absolute lymphocyte count was 540/cu.mm; and the median platelet count was 38000/cu.mm. Table 2 shows the demographic and baseline characteristics of the study cohort with bone marrow infections. 

**Table 1:** Hematological and bone marrow aspirate/biopsy findings

Findings	No. of cases (%)
Hematological findings	
Anemia	29 (100)
Leukopenia	25 (86.2)
Thrombocytopenia	27 (93.1 )
Neutropenia	24 (82.7)
Bone marrow aspirate/biopsy findings	
Increased cellularity	11 (37.9)
Plasmacytosis	13 (44.8)
Lymphocytosis	2 (6.8)
Eosinophilia	6 (20.6)
Increased histiocytes	17 (58.6)
Hemophagocytosis	9 (31)
Erythroid hyperplasia	14 (48.2)
Increased iron stores	7 (24.1)
Leishman-Donovan bodies	19 (65.5)
Granuloma	5 (17.2)
Histoplasma	3 (10.3)
Cryptococcus	1 (3.4)
Special stains positivity	
Ziehl-Neelsen stain	2 (6.8)
Fite-Faraco	1 (3.4)
Periodic acid Schiff, Gomori's methenamine silver	2 (6.8)

**Table 2:** Demographic and baseline characteristics of 29 patients with bone marrow infections

Sl.No	Characteristic	Median	Range
1	Age (years)	30	2-68
2	Hemoglobin (gm/dl)	6.8	3.3-10.9
3	Platelet count (/cu.mm)	38000	3000-207000
4	Total leukocyte count (/cu.mm)	2080	200-17100
5	Absolute neutrophil count (/cu.mm)	780	60-15903
6	Absolute lymphocyte count (/cu.mm)	540	70-2481

Bone Marrow Morphology

On BME, prominence of histiocytes was the most frequently observed finding in patients presenting with fever, organomegaly, and pancytopenia, followed by erythroid hyperplasia and plasmacytosis. In addition, there was evidence of hemophagocytosis in nine cases (31%). The detailed BMA/biopsy findings are represented in Table 1. 

Etiological Spectrum 

Specific infective agents could be identified in 23 cases using Giemsa stain of BMA smears and were highlighted by the relevant special stains either on aspirate or on biopsy sections, which included *Leishmania donovani *(19 cases), *Histoplasma capsulatum* (three cases), and *Cryptococcus neoformans *(one case). The other six required special stains that included ZN and Fite-Faraco stain, which highlighted* Mycobacterium tuberculosis* and *Mycobacterium leprae,* respectively. A triad of fever, splenomegaly, and pancytopenia was strongly associated with leishmaniasis.

**Figure 1:**
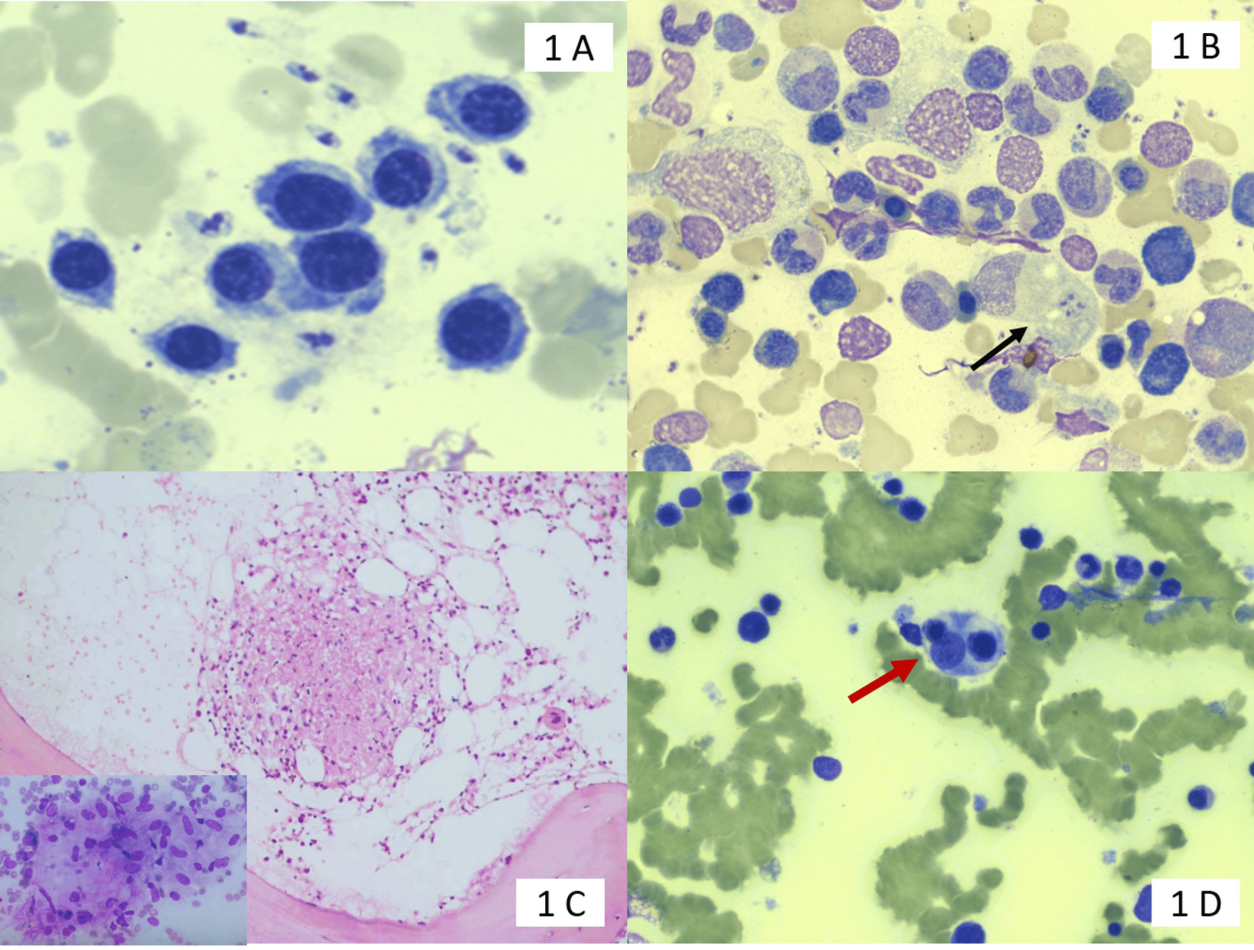
Bone marrow aspiration findings using various stains LD bodies: Leishman-Donovan bodies (A) Bone marrow aspirate smear shows extracellular LD bodies (May-Grunwald-Giemsa, x1000). (B) A bone marrow aspirate smear shows both extracellular and intracellular (marked with black arrow) LD bodies (May-Grunwald-Giemsa, x400). (C) Bone marrow trephine biopsy showing necrotizing epitheloid cell granuloma (hematoxylin and eosin, 400X). The subset image shows an epitheloid cell granuloma in a bone marrow aspirate (hematoxylin and eosin, x400). (D) Bone marrow aspirate smear shows hemophagocytosis by a histiocyte (red arrow) (May-Grunwald-Giemsa, x400)

**Figure 2:**
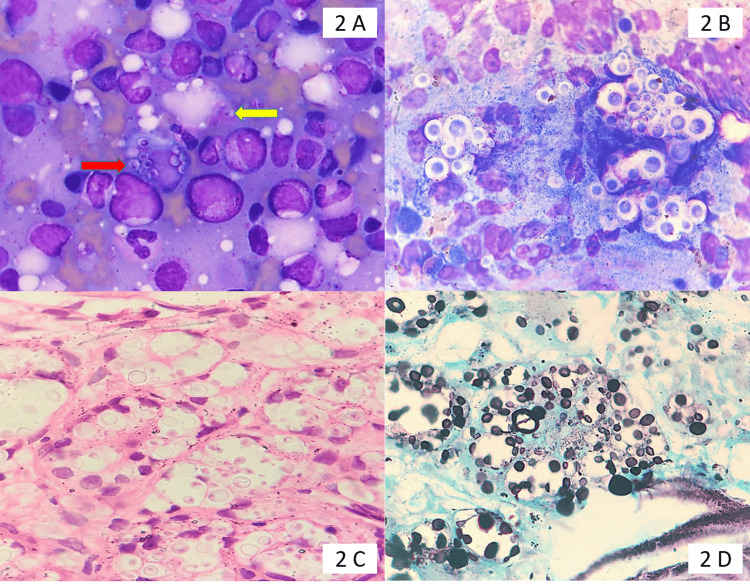
Bone marrow aspiration findings using various stains (A) Bone marrow aspirate smear shows both extracellular (yellow arrow) and intracellular (red arrow) forms of* Histoplasma* sp. These can be identified by an eccentrically placed nucleus and a thick cell wall (May-Grunwald-Giemsa, ×1000). (B) A bone marrow aspirate smear shows numerous yeast forms of *Cryptococcus *identified by a thick capsule (May-Grunwald-Giemsa, ×400). (C) Bone marrow trephine biopsy showing numerous refractile yeast forms surrounded by cystic spaces (hematoxylin and eosin, ×400). (D) Silver methenamine stain on trephine biopsy highlighting the yeast forms of *Cryptococcus *(×400)

A bone marrow aspirate smear showing both extracellular and intracellular Leishman-Donovan (LD) bodies is shown in Figure 1B. A bone marrow trephine biopsy showing a necrotizing epithelioid cell granuloma is shown in Figure 1C. A bone marrow aspirate smear showing hemophagocytosis is shown in Figure 1D. A bone marrow aspirate smear showing both extracellular (yellow arrow) and intracellular (red arrow) forms of *Histoplasma* species is shown in Figure 2A. These can be identified by an eccentrically placed nucleus and a thick cell wall. A bone marrow aspirate smear showing numerous yeast forms of *Cryptococcus*, identified by a thick capsule, is shown in Figure 2B. Bone marrow trephine biopsy showing numerous refractile yeast forms surrounded by cystic spaces, and these yeast forms, highlighted by special stain silver methenamine, are shown in Figures 2C-2D. 

## Discussion

BME is a simple, rapid, reliable, and cheap diagnostic modality to diagnose infections. With the advancement in microbiological laboratory diagnosis for various infectious diseases, the value of BMA is being under-recognized for the diagnosis of infectious pathologies.

The response of bone marrow to various infectious agents results in specific morphological features which aid in the histopathological diagnosis [1]. Bacterial infections lead to hypercellularity of marrow with left shift in granulopoiesis. Prominence of plasma cells with plasma cell satellitosis may be noted with chronic infections. Viral infections may lead to prominence of lymphocytes, plasma cells, and macrophages in the bone marrow [4]. Specific parasitic organisms like *Leishmania donovani *and the malarial parasite can be detected on routine BMA or biopsy preparations. In both visceral leishmaniasis and histoplasmosis, organisms are located within the macrophages, the latter being GMS-positive [5].

Patients with infectious diseases show various hematological alterations as seen in the present study. Anemia was the most common finding with a mean hemoglobin of 6.9 g/dl, followed by thrombocytopenia, pancytopenia, and leukopenia. Similar results were revealed in the studies conducted by Gupta et al. [6].

In the present study, the most common infection diagnosed was leishmaniasis, which comprised 65.5% of the cases, where amastigote forms of LD bodies were seen with the presence of both extracellular and intracellular LD bodies. Fever, anorexia, and hepatosplenomegaly were the most common clinical features in these patients, which is consistent with various studies on visceral leishmaniasis [5-7]. Pancytopenia was the most common peripheral blood finding observed in patients with visceral leishmaniasis in this study, which is in accordance with published reports from nonendemic regions. However, a study by Daneshbod et al. from an endemic region reported pancytopenia in only 10% cases of leishmaniasis [8]. In our study, erythroid hyperplasia was the most prominent finding in BMA, followed by histiocytosis and plasmacytosis. Similar findings were noted in studies by Kumar et al. and Bhatia et al. [1,9]. We also found increased iron stores on Perl’s staining of BMA in 36.8% (7/19) cases of leishmaniasis. The high proportion of leishmaniasis in this study might be due to referral bias, being a tertiary care center, as patients with unexplained cytopenias are more likely to undergo BME.

Out of three cases of tuberculosis in this study, two cases showed epithelioid cell granulomas in BMA, and one case showed granulomas in BMB. However, the ZN stain was positive in two cases only. Despite the availability of effective GeneXpert MTB/Rifampicin (RIF) testing for sputum, Bharuthram et al. had demonstrated that BME continues to be useful [10].

Another unique diagnosis found in this study was disseminated MAC infection in a setting of HIV infection. Disseminated MAC infections are more common in patients with HIV/AIDS, reported in up to 50% of patients [4]. International studies consistently indicate that the prevalence of MAC infection tends to be higher than that of MTB in various regions [10].

In the present study, fungal organisms were detected in four cases (13.7%), of which three cases revealed *Histoplasma capsulatum *and one case of cryptococcosis. Out of three cases of histoplasmosis, two cases showed the presence of intracellular *Histoplasma *organisms, and one showed both intra- and extracellular organisms, which were also positive for special stains, PAS and GMS.

*Cryptococcus neoformans *infection is rare among immunocompetent patients [11]. In this study, cryptococcosis was diagnosed in a three-year-old child. Hematological investigations revealed leukocytosis with marked eosinophilia (AEC: 7712/mm³). BMA showed extensive infiltration with PAS-positive encapsulated yeast forms of *Cryptococcus*. Luo et al. studied 23 pediatric patients with cryptococcosis, and all were non-HIV-infected and immunocompetent children with no specific clinical manifestations. Their case findings are in concordance with our case [12]. This is in contrast to other studies where bone marrow cryptococcosis has been mostly described in patients having concurrent HIV infection [13].

Apart from cases of MTB, two other cases in this study showed a granulomatous reaction. One of them showed epithelioid cell granulomas in both BMA and biopsy with a positive Fite-Faraco stain, revealing involvement of the bone marrow by Hansen's disease. The other case showed epithelioid cell granulomas in BMB along with evidence of hemophagocytosis in a known case of Hansen's disease. However, the Fite stain was negative in this case. Both cases had pancytopenia on peripheral blood examination. Despite the rarity, bone marrow involvement in leprosy should be kept in mind, especially if a known case presents with unexplained cytopenias [14].

The study has some limitations. This study was retrospective in nature and depended on available medical records and slides. Selection bias cannot be excluded, as BME is usually performed in clinically severe or diagnostically challenging cases.

## Conclusions

BME is a simple, rapid, reliable, and cost-effective diagnostic modality in the diagnosis of infections, particularly in patients presenting with PUO and cytopenias. Careful morphological evaluation combined with appropriate special stains can facilitate early diagnosis, especially in resource-limited settings.
